# Supplementary material for: In Vitro Anticancer Activity of Two Ferrocene-Containing Camphor Sulfonamides as Promising Agents against Lung Cancer Cells
Source: Biomedicines. 2022 Jun 8;10(6):1353. doi: 10.3390/biomedicines10061353 (PMC9219647; doi:10.3390/biomedicines10061353)
Supplement: Supplementary file 1 [file biomedicines-10-01353-s001.zip › biomedicines-1756697-supplementary.pdf]

Supplementary Data

Cytotoxic potential of two ferrocene camphor sulfonamides DK-164 and CC-78 tested on lung cancer cell lines

Maria Schröder[1†], Maria Petrova[1†], Zlatina Vlahova[1], Georgi M. Dobrikov[2], Ivailo Slavchev[2], Evdokia Pasheva[1] and Iva Ugrinova[1*]

[1] Institute of Molecular Biology "Akad. Roumen Tsanev", Bulgarian Academy of Sciences, Acad. G. Bonchev str, bl 21, Sofia 1113, Bulgaria

[2] Institute of Organic Chemistry with Center of Phytochemistry, Bulgarian Academy of Sciences, Acad. G. Bonchev str, bl 9, Sofia 1113, Bulgaria

** Correspondence: ugryiva@gmail.com, Tel.: +359 875 117654*
† The authors have equal contribution

1 Chemistry and biology

1.1 General and methods

Chemistry and NMR

For thin layer chromatography (TLC) aluminum sheets pre-coated with silica gel 60 F_{254} (Merck) were used. Flash column chromatography was carried out using silica gel 60 (0.040–0.063 mm, 230-400 mesh ASTM, Merck). Commercially available solvents for reactions, TLC and column chromatography were used after distillation (and were dried if needed) – water, acetic acid (AcOH), methanol (MeOH), petroleum ether (PE), diethyl ether (Et_2O), ethylacetate (EA), dichloromethane (DCM), chloroform, methyl *tert*-butyl ether (MTBE), diisopropyl ether (DIPE), tetrahydrofurane (THF), dimethylformamide (DMF), *iso*-propanol (*i*-PrOH), dimethylsulfoxyde (DMSO). Any fine chemical, whose synthesis is not described or mentioned in the references in this study, is commercially available from Sigma-Aldrich, Fluorochem, Fluka, Acros or Alfa Aesar. Melting temperatures were determined in capillary tubes on an Electrothermal MEL-TEMP 1102D-230 VAC apparatus without corrections. The NMR spectra were recorded on a Bruker Avance II+ 600 spectrometer (600.13 MHz for ^{1}H and 150.92 MHz for ^{13}C). In case of $CDCl_3$, TMS was used as internal standard (δ=0.00). For other deuterated solvents ^{1}H spectra were calibrated to the residual solvent peaks (DMSO-d_6 δ=2.50). ^{13}C spectra were calibrated in all cases to the residual solvent peaks ($CDCl_3$ δ=77.00, DMSO-d_6 δ=39.52). The following additional NMR techniques were used for all compounds: DEPT 135, COSY, HSQC and HMBC. ^{1}H and ^{13}C NMR data are reported as follows: chemical shift (in ppm), multiplicity (s = singlet, d = doublet, t = triplet, q = quartet, br = broad, m = multiplet), integration, identification, and coupling constants (in Hz). Singlets in ^{13}C NMR spectra were not mentioned with "s" in all cases. Mass spectra (MS) were recorded on a Shimadzu Liquid Chromatograph Mass Spectrometer LCMS-2020 and Waters ZQ 2000. Direct MS-regime was applied; each compound was dissolved in MeOH/formic acid (1% or 10%) with concentration 1 mg/ml; MS detector with electrospray ionization (ESI). MS spectra are reported as fragmentation in m/z with relative intensities (%).The compound names are in agreement with the IUPAC nomenclature.

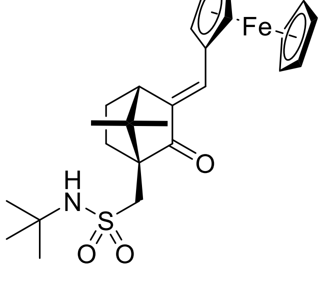

Figure S1. Compound DK-164

Biology

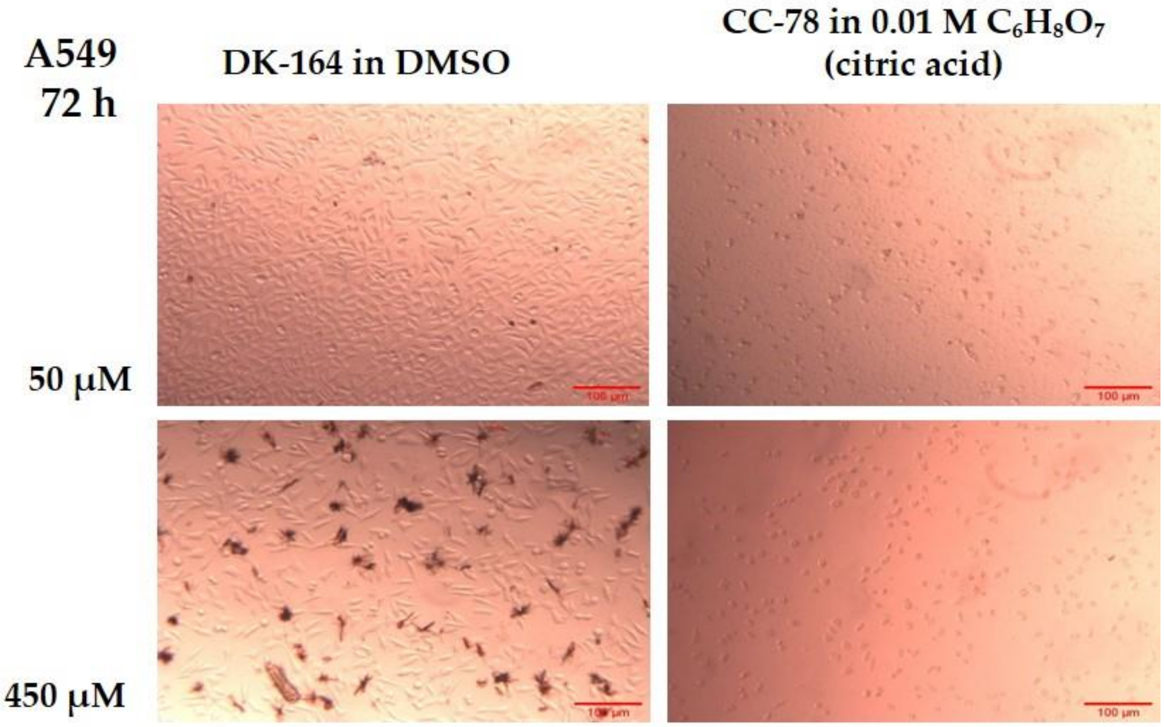

Figure S2. Microscopic images of A549 lung cancer cells treated with DK-164 (diluted in DMSO) and CC-78 (diluted in 0.01 M Citric acid) for 72h.
As the concentration of DK-164 increased, crystal formation was observed.

1.2 Synthesis of target compounds

1.2.1 Synthesis of 1-((1*S*,4*R*)-7,7-dimethyl-2-oxobicyclo[2.2.1]heptan-1-yl)-*N*-(2-hydroxynaphthalen-1-yl)methanesulfonamide (**8**)

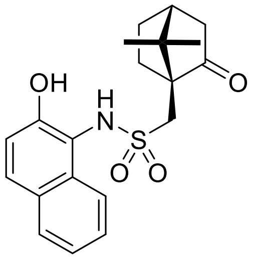

In 20 ml dry DCM were dissolved 0.254 g (1.60 mmol, 1.0 equiv.) of 1-aminonaphthalen-2-ol (**2**) and 0.33 ml (2.39 mmol, 1.5 equiv.) Et_3N. The solution was cooled to 0°C and 0.400 g (1.60 mmol, 1.0 equiv.) of (1*S*)-(+)-10-camphorsulfonyl chloride (**1**) was added. The mixture was stirred for 18 h at r.t. The reaction progress was monitored by TLC (DCM). Workup: the mixture was washed with aq. citric acid and water, then dried over anhydr. Na_2SO_4 and evaporated to dryness. Column chromatography: DCM. After column the product was boiled with PE, cooled, decanted and dried *in vacuo* to give 0.380 g (64%) of pure **8** as beige crystals. M.p. 138-139°C. ^{1}H NMR (600.13 MHz, $CDCl_3$, 298 K): δ = 7.78–7.83 (m, 2H, CH_{arom}), 7.46–7.51 (m, 2H, CH_{arom}), 7.42 (d, 1H, CH_{arom}, *J* = 8.9 Hz), 7.27 (d, 1H, CH_{arom}, *J* = 8.9 Hz), 4.66 (br s, 2H, OH, NH), 3.92 (d, 1H, CH_2SO_2, *J* = 14.9 Hz), 3.30 (d, 1H, CH_2SO_2, *J* = 14.9 Hz), 2.51 (m, 1H, CH_2), 2.44 (m, 1H, CH_2.), 2.16 (m, 1H, CH), 2.09 (m, 1H, CH_2), 2.00 (d, 1H, CH_2, *J* = 18.6 Hz), 1.86 (ddd, 1H, CH_2, *J* = 14.1, 9.4, 4.8 Hz), 1.48 (ddd, 1H, CH_2, *J* = 13.0, 9.4, 4.0 Hz), 1.13 (s, 3H, CH_3), 0.92 (s, 3H, CH_3). ^{13}C NMR (150.92 MHz, $CDCl_3$, 298 K): δ = 214.43 (1C, C=O), 134.71 (1C, quaternary C_{arom}), 132.75 (1C, quaternary C_{arom}), 131.63 (1C, quaternary C_{arom}), 128.58 (1C, CH_{arom}), 126.15 (1C, CH_{arom}), 125.49 (1C, CH_{arom}), 124.25 (1C, quaternary C_{arom}), 121.49 (1C, CH_{arom}), 121.07 (1C, CH_{arom}), 118.44 (1C, CH_{arom}), 58.40 (1C, quaternary $C_{aliphat}$), 48.78 (1C, CH_2SO_2), 48.21 (1C, quaternary $C_{aliphat}$), 42.82 (1C, >CH), 42.52 (1C, >CH_2), 26.89 (1C, >CH_2), 25.30 (1C, >CH_2), 19.79 (1C, CH_3), 19.71 (1C, CH_3) ppm. MS (ESI+) m/z (rel. int.): 769 (83, $[2M+Na]^+$), 428 (100, $[M+MeOH+Na]^+$), 374 (93, $[M+H]^+$). Anal. calcd. for $C_{20}H_{23}NO_4S$ (373.47): C, 64.32; H, 6.21; N, 3.75; S, 8.58. Found: C, 64.38; H, 6.28; N, 3.70; S, 8.54%.

1.2.2 Synthesis of 1-((1*S*,4*R*)-7,7-dimethyl-2-oxobicyclo[2.2.1]heptan-1-yl)-*N*-(2,4-dioxo-1,2,3,4-tetrahydropyrimidin-5-yl)methanesulfonamide (**9**)

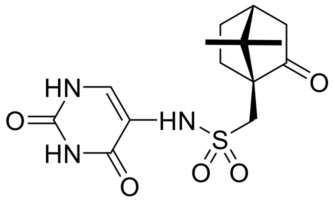

In 5 ml dry pyridine were suspended 0.296 g (2.33 mmol, 1.0 equiv.) of 5-aminouracil (**3**) and (1*S*)-(+)-10-camphorsulfonyl chloride (3.50 mmol, 1.5 equiv.) (**1**) was added at once. The resulting suspension was stirred for 96 h at r.t, until the mixture turned into a clear red solution. The solvent was evaporated *in vacuo* and 2N aq. HCl was added and stirred for 2 h. The formed pink suspension was filtered and washed with multiple portions of water. The solid was dried in desiccator for 24 h, washed with hot MTBE and dried *in vacuo* to give 0.587 g (74%) of pure **9** as pale pink powder. M.p. 214-215°C. ^{1}H NMR (600.13 MHz, DMSO-d_6, 298 K): δ = 11.41 (br d, 1H, heterocyclic CO-NH-CO, *J* = 1.8 Hz), 11.01 (dd, 1H, heterocyclic =CH-NH, *J* = 6.0, 1.8 Hz), 8.81 (s, 1H, SO_2NH), 7.43 (d, 1H, heterocyclic =CH, *J* = 6.0 Hz), 3.40 (d, 1H, CH_2SO_2, *J* = 15.1 Hz), 3.09 (d, 1H, CH_2SO_2, *J* = 15.1 Hz), 2.30–2.39 (m, 2H, CH_2), 2.04 (m, 1H, >CH), 1.88–1.95 (m, 2H, CH_2), 1.51 (ddd, 1H, CH_2, *J* = 13.9, 9.4, 4.7 Hz), 1.38 (m, 1H, CH_2), 0.99 (s, 3H, CH_3). 0.77 (s, 3H, CH_3) ppm. ^{13}C NMR (150.92 MHz, DMSO-d^6, 298 K): δ = 214.47 (1C, aliphatic C=O), 162.16 (1C, heterocyclic C=O), 150.58 (1C, heterocyclic C=O), 139.74 (1C, heterocyclic =CH), 110.60 (1C, quaternary =C-NH), 58.08 (1C, quaternary $C_{aliphat}$), 49.64 (1C, CH_2SO_2), 47.59 (1C, quaternary $C_{aliphat}$), 42.05 (2C, >CH_2 and >CH), 26.28 (1C, >CH_2), 24.69 (1C, >CH_2), 19.58 (1C, CH_3), 19.39 (1C, CH_3). MS (ESI+) m/z (rel. int.): 705 (36, $[2M+Na]^+$), 396 (100, $[M+MeOH+Na]^+$), 364 (61, $[M+Na]^+$), 342 (10, $[M+H]^+$). Anal. calcd. for $C_{14}H_{19}N_3O_5S$ (341.38): C, 49.26; H, 5.61; N, 12.31; S, 9.39. Found: C, 49.33; H, 5.58; N, 12.35; S, 9.36%.

1.2.3 Synthesis of *N*-(2-(1*H*-indol-3-yl)ethyl)-1-((1*S*,4*R*)-7,7-dimethyl-2-oxobicyclo[2.2.1]heptan-1-yl)methanesulfonamide (**10**)

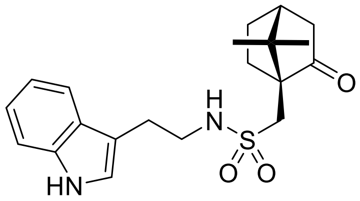

In 20 ml dry DCM were dissolved 0.410 g (2.08 mmol, 1.1 equiv.) of tryptamine (**4**) and 0.66 ml (4.74 mmol, 2.5 equiv.) Et_3N. The solution was cooled to 0°C and 0.475 g (1.90 mmol, 1.0 equiv.) of (1*S*)-(+)-10-camphorsulfonyl chloride (**1**) were added. The mixture was stirred for 1h at 0 °C and 72 h at r.t. The reaction progress was monitored by TLC (DCM/MTBE = 50/1). The reaction mixture was washed successively with aqueous citric acid and water, dried over $MgSO_4$, and evaporated to dryness. The crude product was purified over silica gel (DCM/MTBE = 50/1) to afford the title compound **10** (0.660 g, 93%) as a pale yellow thick oil. ^{1}H NMR (600.01 MHz, $CDCl_3$, 293 K): δ = 8.09 (s, 1H, heterocyclic N$\underline{H}$), 7.58–7.63 (m, 1H, heterocyclic C$\underline{H}$), 7.34–7.39 (m, 1H, heterocyclic C$\underline{H}$), 7.17–7.22 (m, 1H, heterocyclic C$\underline{H}$), 7.09–7.14 (m, 2H, heterocyclic C$\underline{H}$), 5.08 (s, 1H, $SO_2N\underline{H}$), 3.43–3.56 (m, 2H, $C\underline{H}_2$), 3.32 (d, 1H, $C\underline{H}_2SO_2$, *J* = 15.1 Hz), 3.01–3.14 (m, 2H, $C\underline{H}_2$), 2.82 (d, 1H, $C\underline{H}_2SO_2$, *J* = 15.1 Hz), 2.26–2.34 (m, 1H, $C\underline{H}_2$), 2.15–2.23 (m, 1H, $C\underline{H}_2$), 2.07 (m, 1H, C$\underline{H}$), 1.93–2.02 (m, 1H, $C\underline{H}_2$), 1.86 (d, 1H, $C\underline{H}_2$, *J* = 18.5 Hz), 1.78–1.84 (m, 1H, $C\underline{H}_2$), 1.34–1.41 (m, 1H, $C\underline{H}_2$), 0.97 (s, 3H, $C\underline{H}_3$), 0.76 (s, 3H, $C\underline{H}_3$) ppm. ^{13}C NMR (150.87 MHz, $CDCl_3$, 293 K): δ = 216.55 (1C, $\underline{C}$=O), 136.54 (1C, quaternary C_{arom}), 127.20 (1C, quaternary C_{arom}), 122.74 (1C, $\underline{C}H_{arom}$), 122.36 (1C, $\underline{C}H_{arom}$), 119.68 (1C, $\underline{C}H_{arom}$), 118.78 (1C, $\underline{C}H_{arom}$), 112.21 quaternary C_{arom}), 111.40 (1C, $\underline{C}H_{arom}$), 59.10 (1C, quaternary $C_{aliphat}$), 49.32 (1C, $\underline{C}H_2$), 48.69 (1C, quaternary $C_{aliphat}$), 43.86 (1C, $\underline{C}H_2$), 42.96 (1C, $\underline{C}H_2$), 42.82 (1C, $\underline{C}$H), 27.10 (1C, $\underline{C}H_2$), 26.39 (1C, $\underline{C}H_2$), 26.21 (1C, $\underline{C}H_2$), 19.88 (1C, $\underline{C}H_3$), 19.62 (1C, $\underline{C}H_3$). MS (ESI+) m/z (rel. int.): 771 (67, $[2M+Na]^+$), 429 (22, $[M+MeOH+Na]^+$), 397 (100, $[M+Na]^+$), 375 (49, $[M+H]^+$). Anal. calcd. for $C_{20}H_{26}N_2O_3S$ (374.50): C, 64.14; H, 7.00; N, 7.48; S, 8.56. Found: C, 64.10; H, 7.06; N, 7.42; S, 8.53%.

1.2.4 Synthesis of 1-((1*S*,4*R*)-7,7-dimethyl-2-oxobicyclo[2.2.1]heptan-1-yl)-*N*-(1,3,4-thiadiazol-2-yl)methanesulfonamide (**11**)

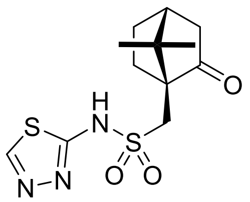

In 20 ml dry DCM were dissolved 0.202 g (2.0 mmol, 1.0 equiv.) of 1,3,4-thiadiazol-2-amine (**5**) and 0.36 ml (2.6 mmol, 1.3 equiv.) Et_3N. The solution was cooled to 0°C and 0.500 g (2.0 mmol, 1.0 equiv.) of (1*S*)-(+)-10-camphorsulfonyl chloride (**1**) were added. The mixture was stirred for 1h at 0 °C and 24 h at r.t. The reaction progress was monitored by TLC (DCM/MTBE = 5/3). The reaction mixture was washed with water, dried over Na_2SO_4 and evaporated to dryness. The crude product was purified over silica gel (DCM/MTBE = 10/1; 5:3) to afford the title compound **11** (0.096 g, 15%) as light yellow crystals. M.p. 198-199°C. ^{1}H NMR (600.01 MHz, $CDCl_3$, 293 K): δ = 11.75 (br. s, 1H, $SO_2N\underline{H}$), 8.24 (s, 1H, heterocyclic C$\underline{H}$), 3.56 (d, 1H, $C\underline{H}_2SO_2$, *J* = 14.8 Hz), 3.04 (d, 1H, $C\underline{H}_2SO_2$, *J* = 14.8 Hz), 2.49–2.58 (m, 1H, $C\underline{H}_2$), 2.30–2.41 (m, 1H, $C\underline{H}_2$), 2.09–2.14 (m, 1H, C$\underline{H}$), 2.01–2.09 (m, 1H, $C\underline{H}_2$), 1.95 (d, 1H, $C\underline{H}_2$, *J* = 18.5 Hz), 1.73–1.82 (m, 1H, $C\underline{H}_2$), 1.39–1.48 (m, 1H, $C\underline{H}_2$), 1.10 (s, 3H, $C\underline{H}_3$), 0.86 (s, 3H, $C\underline{H}_3$) ppm. ^{13}C NMR (150.87 MHz, $CDCl_3$, 293 K): δ = 216.18 (1C, $\underline{C}$=O), 167.78 (1C, quaternary $\underline{C}_{heteroarom}$), 142.89 (1C, $\underline{C}H_{heteroarom}$), 58.60 (1C, quaternary $C_{aliphat}$), 50.36 (1C, $\underline{C}H_2$), 48.61 (1C, quaternary $C_{aliphat}$), 42.91 (1C, $\underline{C}H_2$), 42.82 (1C, $\underline{C}$H), 27.22 (1C, $\underline{C}H_2$), 24.85 (1C, $\underline{C}H_2$), 20.03 (1C, $\underline{C}H_3$), 20.00 (1C, $\underline{C}H_3$). MS (ESI+) m/z (rel. int.): 653 (39, $[2M+Na]^+$), 631 (19, $[2M+H]^+$), 370 (32, $[M+MeOH+Na]^+$), 316 (64, $[M+H]^+$). Anal. calcd. for $C_{12}H_{17}N_3O_3S_2$ (315.41): C, 45.70; H, 5.43; N, 13.32; S, 20.33. Found: C, 45.77; H, 5.40; N, 13.25; S, 20.27%.

1.2.5 Synthesis of (1*S*,4*R*)-1-(((4-(2-(dimethylamino)ethyl)piperazin-1-yl)sulfonyl)methyl)-7,7-dimethylbicyclo[2.2.1]heptan-2-one (**12**)

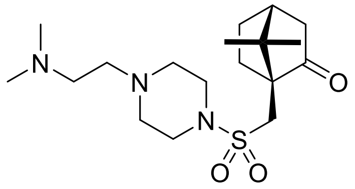

In 20 ml dry DCM were dissolved 0.408 g (2.6 mmol, 1.3 equiv.) of ethyl N,N-dimethyl-2-(piperazin-1-yl)ethan-1-amine (**6**), 0.555 ml (4.0 mmol, 2.0 equiv.) of Et_3N, and 0.5 g (2.0 mmol, 1.0 equiv.) of (1*S*)-(+)-10-camphorsulfonyl chloride (**1**). After stirring for 24 h at r.t. the reaction mixture was diluted with water and extracted with DCM. The combined organic extracts were dried and the solvent was removed under reduced pressure. The crude product was filtered through silica pad (acetone:Et_3N=100:1 as eluent) and evaporated. The product was treated with petroleum ether and the resulting mixture was stored in the fridge overnight to form white solid. This solid was dried *in vacuo* to give white crystals of pure **12** (quantitative yield). ^1H NMR (600.01 MHz, $CDCl_3$, 293 K): δ = 3.25-3.38 (m, 5H, $C\underline{H}_2$), 2.70 (d, 1H, $C\underline{H}_2SO_2$, J = 14.5 Hz), 2.53–2.58 (m, 4H, $C\underline{H}_2$), 2.45–2.53 (m, 3H, $C\underline{H}_2$), 2.39–2.45 (m, 2H, $C\underline{H}_2$), 2.31–2.39 (m, 1H, $C\underline{H}_2$), 2.24 (s, 6H, $N(C\underline{H}_3)_2$), 2.06–2.10 (m, 1H, $C\underline{H}$), 1.98–2.06 (m, 1H, $C\underline{H}_2$), 1.92 (d, 1H, $C\underline{H}_2$, J = 18.5 Hz), 1.58–1.68 (m, 1H, $C\underline{H}_2$), 1.35–1.45 (m, 1H, $C\underline{H}_2$), 1.10 (s, 3H, $C\underline{H}_3$), 0.85 (s, 3H, $C\underline{H}_3$) ppm. ^{13}C NMR (150.87 MHz, $CDCl_3$, 293 K): δ = 215.21 (1C, $\underline{C}$=O), 58.21 (1C, quaternary C), 56.83 (1C, $\underline{C}H_2$), 56.30 (1C, $\underline{C}H_2$), 53.10 (2C, $\underline{C}H_2$), 47.98 (1C, quaternary C), 45.96 (2C, $N(\underline{C}H_3)_2$), 45.78 (2C, $\underline{C}H_2$), 44.26 (1C, $\underline{C}H_2$), 42.86 (1C, CH), 42.67 (1C, $\underline{C}H_2$), 27.01 (1C, $\underline{C}H_2$), 25.18 (1C, $\underline{C}H_2$), 20.11 (1C, $\underline{C}H_3$), 19.85 (1C, $\underline{C}H_3$). MS (ESI+) m/z (rel. int.): 372 (M+H, 100), 386 (M-Me, 57). Anal. calcd. for $C_{18}H_{33}N_3O_3S$ (371.54): C, 58.19; H, 8.95; N, 11.31; S, 8.63. Found: C, 58.26; H, 8.99; N, 11.25; S, 8.60%.

1.2.6 Synthesis of ethyl 1-((((1*S*,4*R*)-7,7-dimethyl-2-oxobicyclo[2.2.1]heptan-1-yl)methyl)sulfonyl)piperidine-4-carboxylate (**13**)

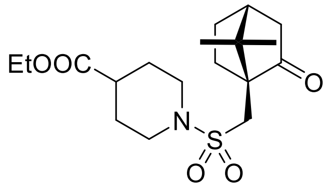

In 20 ml dry DCM were dissolved 1.254 g (8.0 mmol, 1.0 equiv.) of ethyl piperidine-4-carboxylate (**7**), 1.67 ml (12.0 mmol, 1.5 equiv.) of Et_3N, and 2.0 g (8.0 mmol, 1.0 equiv.) of (1*S*)-(+)-10-camphorsulfonyl chloride (**1**). After stirring for 24 h at r.t. the reaction mixture was diluted with water and extracted with DCM. The combined organic extracts were dried and evaporated, and the residue purified over silica gel (DCM; DCM/MTBE = 100/1, 50/1) to afford 2.10 g (71%) of the title compound **13** as white crystals. M.p. 80-81°C. ^{1}H NMR (600.01 MHz, $CDCl_3$, 293 K): δ = 4.15 (q, 2H, $CH_3C\underline{H}_2$, J = 7.1 Hz), 3.66–3.79 (m, 2H, $C\underline{H}_2$), 3.31 (d, 1H, $C\underline{H}_2SO_2$, J = 14.6 Hz), 2.86–3.00 (m, 2H, $C\underline{H}_2$), 2.72 (d, 1H, $C\underline{H}_2SO_2$, J = 14.6 Hz), 2.48–2.56 (m, 1H, $C\underline{H}_2$), 2.39–2.46 (m, 1H, $C\underline{H}$), 2.33–2.39 (m, 1H, $C\underline{H}_2$), 2.08–2.11 (m, 1H, $C\underline{H}$), 1.96–2.08 (m, 3H, $C\underline{H}_2$), 1.93 (d, 1H, $C\underline{H}_2$, J = 18.5 Hz), 1.76–1.86 (m, 2H, $C\underline{H}_2$), 1.56–1.65 (m, 1H, $C\underline{H}_2$), 1.38–1.45 (m, 1H, $C\underline{H}_2$), 1.26 (t, 3H, $C\underline{H}_3CH_2$, J = 7.1 Hz), 1.12 (s, 3H, $C\underline{H}_3$), 0.87 (s, 3H, $C\underline{H}_3$) ppm. ^{13}C NMR (150.87 MHz, $CDCl_3$, 293 K): δ = 215.58 (1C, $\underline{C}$=O), 174.12 (1C, $\underline{C}O_2Et$), 60.83 (1C, $CH_3\underline{C}H_2$), 58.35 (1C, quaternary C), 48.08 (1C, quaternary C), 45.20 (1C, $\underline{C}H_2$), 45.19 (1C, $\underline{C}H_2$), 45.06 (1C, $\underline{C}H_2$), 42.89 (1C, $\underline{C}H$), 42.72 (1C, $\underline{C}H_2$), 40.38 (1C, $\underline{C}H$), 27.99 (2C, $\underline{C}H_2$), 27.04 (1C, $\underline{C}H_2$), 25.18 (1C, $\underline{C}H_2$), 20.13 (1C, $\underline{C}H_3$), 19.91 (1C, $\underline{C}H_3$), 14.32 (1C, $\underline{C}H_3CH_2$). Anal. calcd. for $C_{18}H_{29}NO_5S$ (371.49): C, 58.20; H, 7.87; N, 3.77; S, 8.63. Found: C, 58.15; H, 7.82; N, 3.81; S, 8.67%.

1.2.7 Synthesis of (1*S*,4*S*)-3-((*E*)-ferrocenylmethylene)-1-(((4-(2-(dimethylamino)ethyl)piperazin-1-yl)sulfonyl)methyl)-7,7-dimethylbicyclo[2.2.1]heptan-2-one (**12a**)

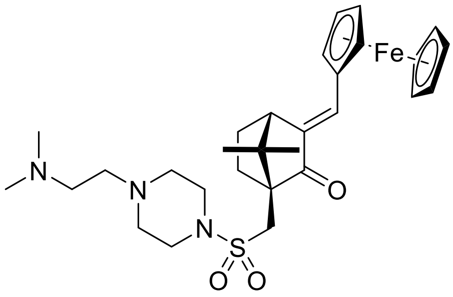

In 8 ml dry DMSO were added consequently 0.400 g (1.08 mmol, 1.0 eq.) **12**, 0.419 g (2.16 mmol, 2.0 eq.) ferrocene carbaldehyde (**14**) and 0.0364 g (0.32 mmol, 0.3 eq.) KOBu-*t* and stirred at r.t. for 3.5 h. Water was added and extracted with Et_2O. Organic phase was washed with water, dried over anhydr. Na_2SO_4 and evaporated. TLC – acetone:Et_3N = 150:1. Crude product was purified by column chromatography – 1) *i*-PrOH; 2) acetone; 3) acetone:Et_3N = 100:1 – for product. After column the product was washed with hot PE and dried *in vacuo* to give 0.347 g (57%) of pure **12a** as dark red crystals. M.p. 118-119°C. ^{1}H NMR (600.01 MHz, $CDCl_3$, 293 K): δ = 7.10 (s, 1H, Fc-C<u>H</u>=C), 4.46–4.53 (m, 2H, ferrocene C<u>H</u>), 4.39–4.45 (m, 2H, ferrocene C<u>H</u>), 4.14 (s, 5H, Cp), 3.45 (d, 1H, C<u>H</u>$_2$SO$_2$, *J* = 14.5 Hz), 3.31–3.42 (m, 4H, C<u>H</u>$_2$), 2.91 (d, 1H, C<u>H</u>, *J* = 4.14 Hz), 2.81 (d, 1H, C<u>H</u>$_2$SO$_2$, *J* = 14.5 Hz), 2.50–2.63 (m, 7H), 2.40–2.49 (m, 2H, C<u>H</u>$_2$), 2.27 (s, 6H, N(C<u>H</u>$_3$)$_2$), 2.14–2.22 (m, 1H, C<u>H</u>$_2$), 1.66–1.74 (m, 1H, C<u>H</u>$_2$), 1.50–1.57 (m, 1H, C<u>H</u>$_2$), 1.16 (s, 3H, C<u>H</u>$_3$), 0.84 (s, 3H, C<u>H</u>$_3$) ppm. ^{13}C NMR (150.87 MHz, $CDCl_3$, 293 K): δ = 203.68 (1C, <u>C</u>=O), 136.52 (1C, quaternary Fc-CH=<u>C</u>), 130.14 (1C, Fc-<u>C</u>H=C), 78.43 (1C, ferrocene quaternary C), 71.76 (1C, ferrocene <u>C</u>H), 71.02 (2C, ferrocene <u>C</u>H), 69.58 (5C, Cp), 69.15 (1C, ferrocene <u>C</u>H), 57.58 (1C, quaternary C), 56.90 (1C, <u>C</u>H$_2$), 56.37 (1C, <u>C</u>H$_2$), 53.19 (2C, CH$_2$), 49.24 (1C, <u>C</u>H), 47.41 (1C, quaternary C), 46.01 (2C, N(<u>C</u>H$_3$)$_2$), 45.86 (2C, <u>C</u>H$_2$), 44.51 (1C, <u>C</u>H$_2$), 26.17 (1C, <u>C</u>H$_2$), 25.72 (1C, <u>C</u>H$_2$), 20.75 (1C, <u>C</u>H$_3$), 19.58 (1C, <u>C</u>H$_3$). MS (ESI+) m/z (rel. int.): 568 (M+1, 100). Anal. calcd. for $C_{29}H_{41}FeN_3O_3S$ (567.57): C, 61.37; H, 7.28; Fe, 9.84; N, 7.40; S, 5.65. Found: C, 61.30; H, 7.36; Fe, 9.75; N, 7.47; S, 5.60%.

2. ^{1}H and ^{13}C NMR spectra of synthesized compounds

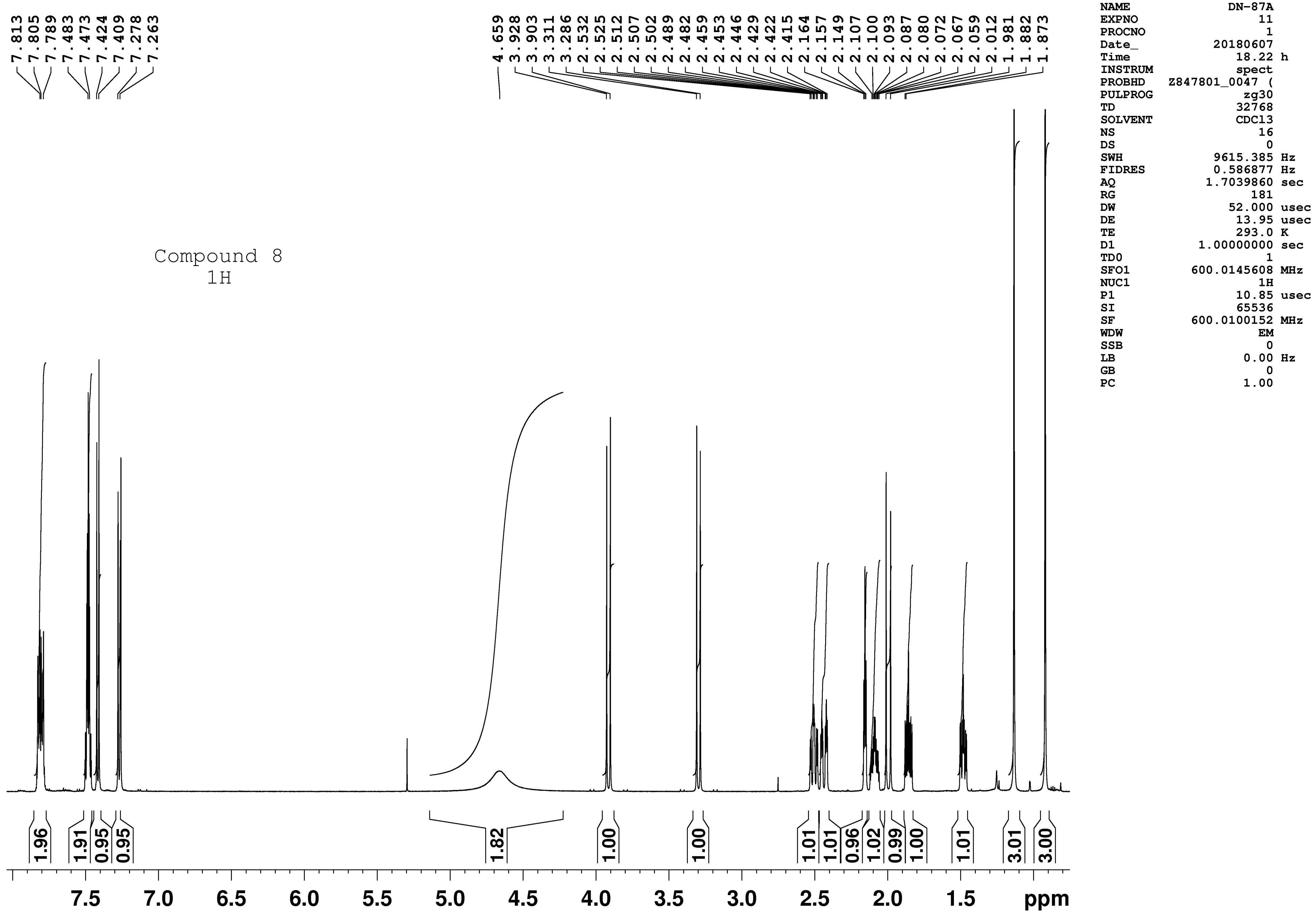
Compound 8
1H
7.813
7.805
7.789
7.483
7.473
7.424
7.409
7.278
7.263
4.659
3.928
3.903
3.311
3.286
2.532
2.525
2.512
2.507
2.502
2.489
2.482
2.459
2.453
2.446
2.429
2.422
2.415
2.164
2.157
2.149
2.107
2.100
2.093
2.087
2.080
2.072
2.067
2.059
2.012
1.981
1.882
1.873
1.96
1.91
0.95
0.95
1.82
1.00
1.00
1.01
1.01
0.96
1.02
0.99
1.00
1.01
3.01
3.00
7.5
7.0
6.5
6.0
5.5
5.0
4.5
4.0
3.5
3.0
2.5
2.0
1.5
ppm
NAME DN-87A
EXPNO 11
PROCNO 1
Date_ 20180607
Time 18.22 h
INSTRUM spect
PROBHD Z847801_0047 (
PULPROG zg30
TD 32768
SOLVENT CDCl3
NS 16
DS 0
SWH 9615.385 Hz
FIDRES 0.586877 Hz
AQ 1.7039860 sec
RG 181
DW 52.000 usec
DE 13.95 usec
TE 293.0 K
D1 1.00000000 sec
TD0 1
SFO1 600.0145608 MHz
NUC1 1H
P1 10.85 usec
SI 65536
SF 600.0100152 MHz
WDW EM
SSB 0
LB 0.00 Hz
GB 0
PC 1.00

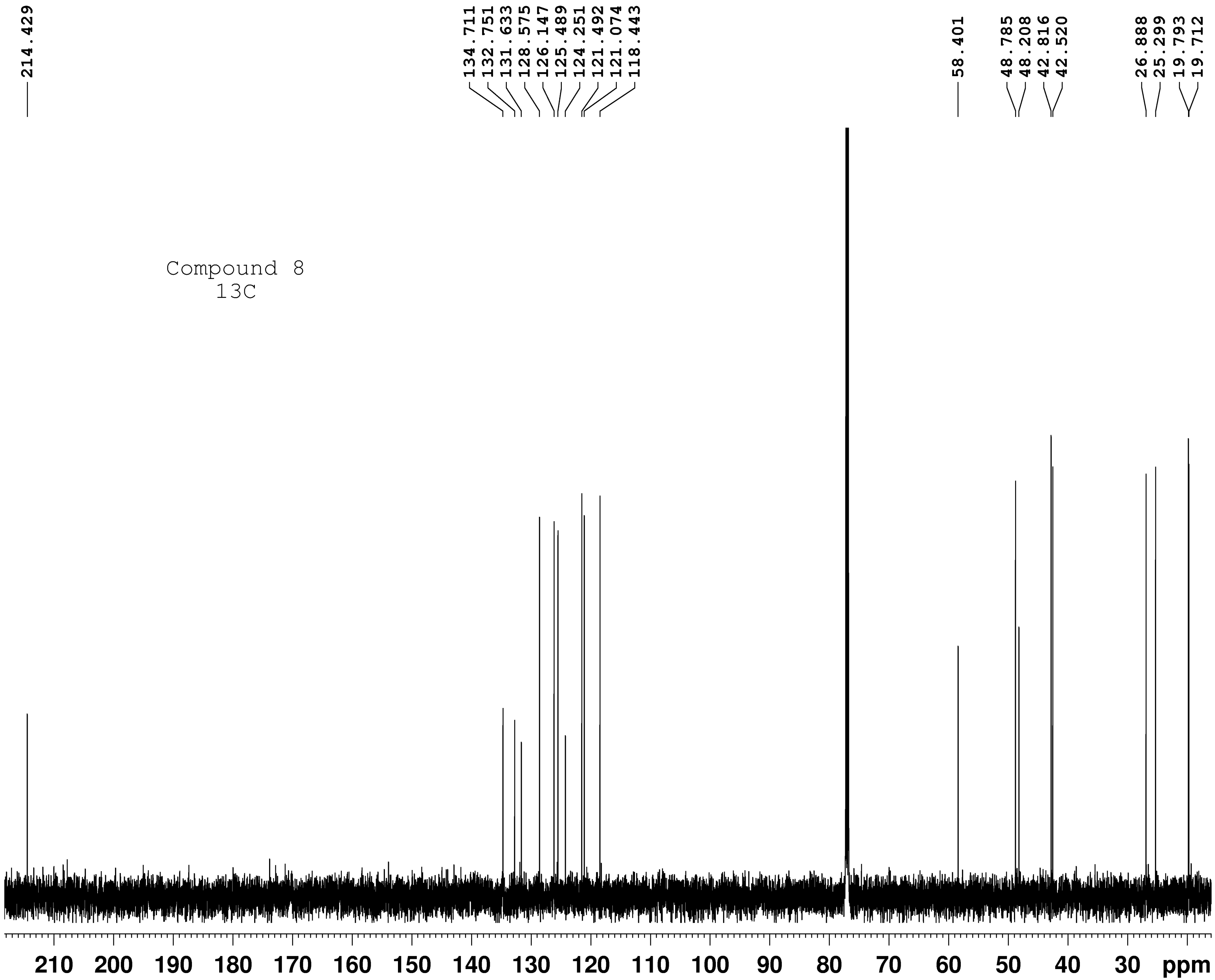

NAME	DN-87A	
EXPNO	12	
PROCNO	1	
Date_	20180607	
Time	18.27	h
INSTRUM	spect	
PROBHD	Z847801_0047 (	
PULPROG	zgdc30	
TD	32768	
SOLVENT	CDCl3	
NS	128	
DS	0	
SWH	36057.691	Hz
FIDRES	2.200787	Hz
AQ	0.4544329	sec
RG	2050	
DW	13.867	usec
DE	6.50	usec
TE	293.0	K
D1	1.50000000	sec
D11	0.03000000	sec
TD0	1	
SFO1	150.8892338	MHz
NUC1	13C	
P1	9.80	usec
SI	65536	
SF	150.8726425	MHz
WDW	EM	
SSB	0	
LB	1.00	Hz
GB	0	
PC	1.40	

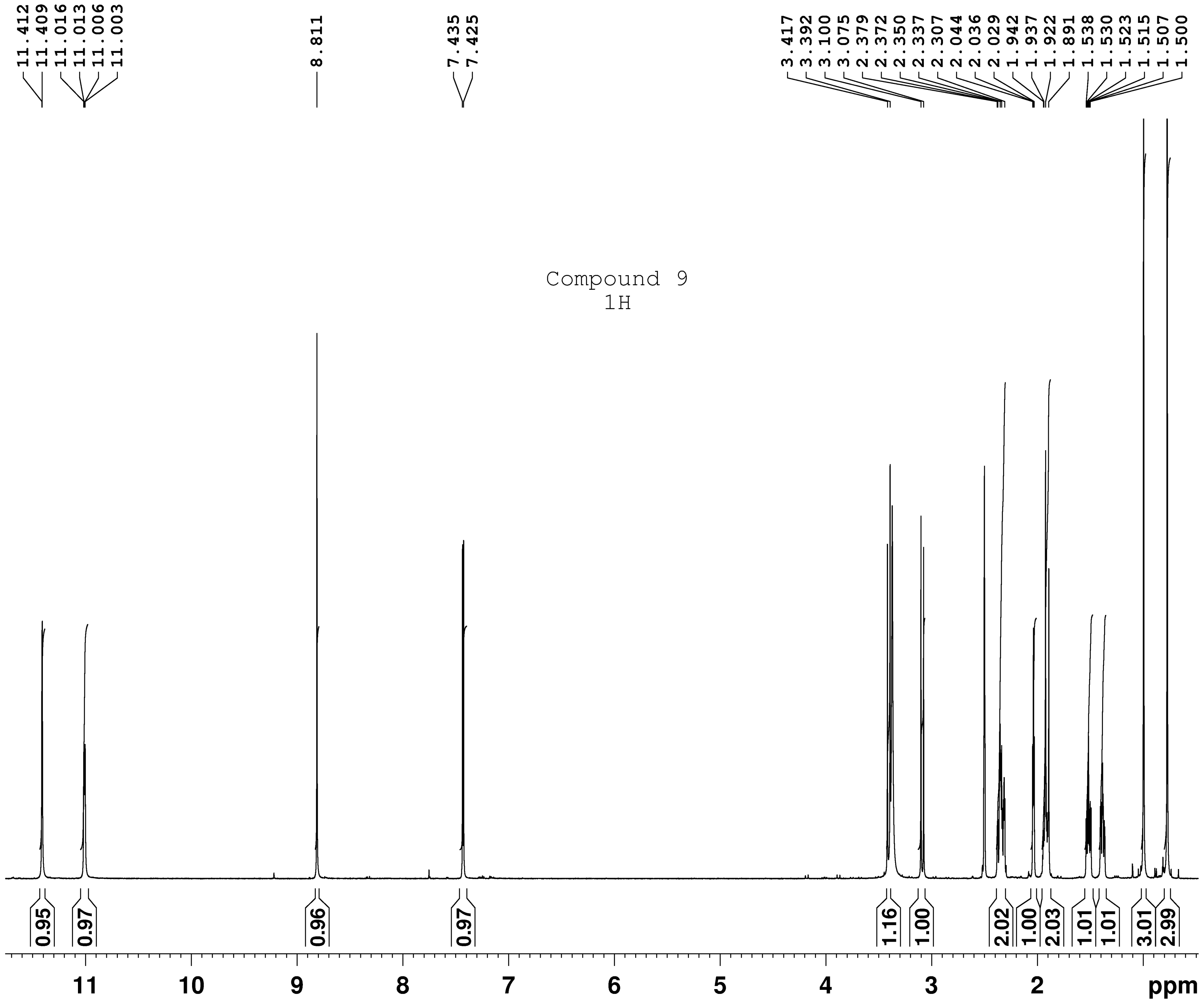

NAME	DN-98A	
EXPNO	11	
PROCNO	1	
Date_	20180629	
Time	14.57	h
INSTRUM	spect	
PROBHD	Z847801_0047 (	
PULPROG	zg30	
TD	32768	
SOLVENT	DMSO	
NS	16	
DS	0	
SWH	9615.385	Hz
FIDRES	0.586877	Hz
AQ	1.7039860	sec
RG	161	
DW	52.000	usec
DE	13.95	usec
TE	293.0	K
D1	1.00000000	sec
TD0	1	
SFO1	600.0145608	MHz
NUC1	1H	
P1	10.85	usec
SI	65536	
SF	600.0100048	MHz
WDW	EM	
SSB	0	
LB	0.00	Hz
GB	0	
PC	1.00	

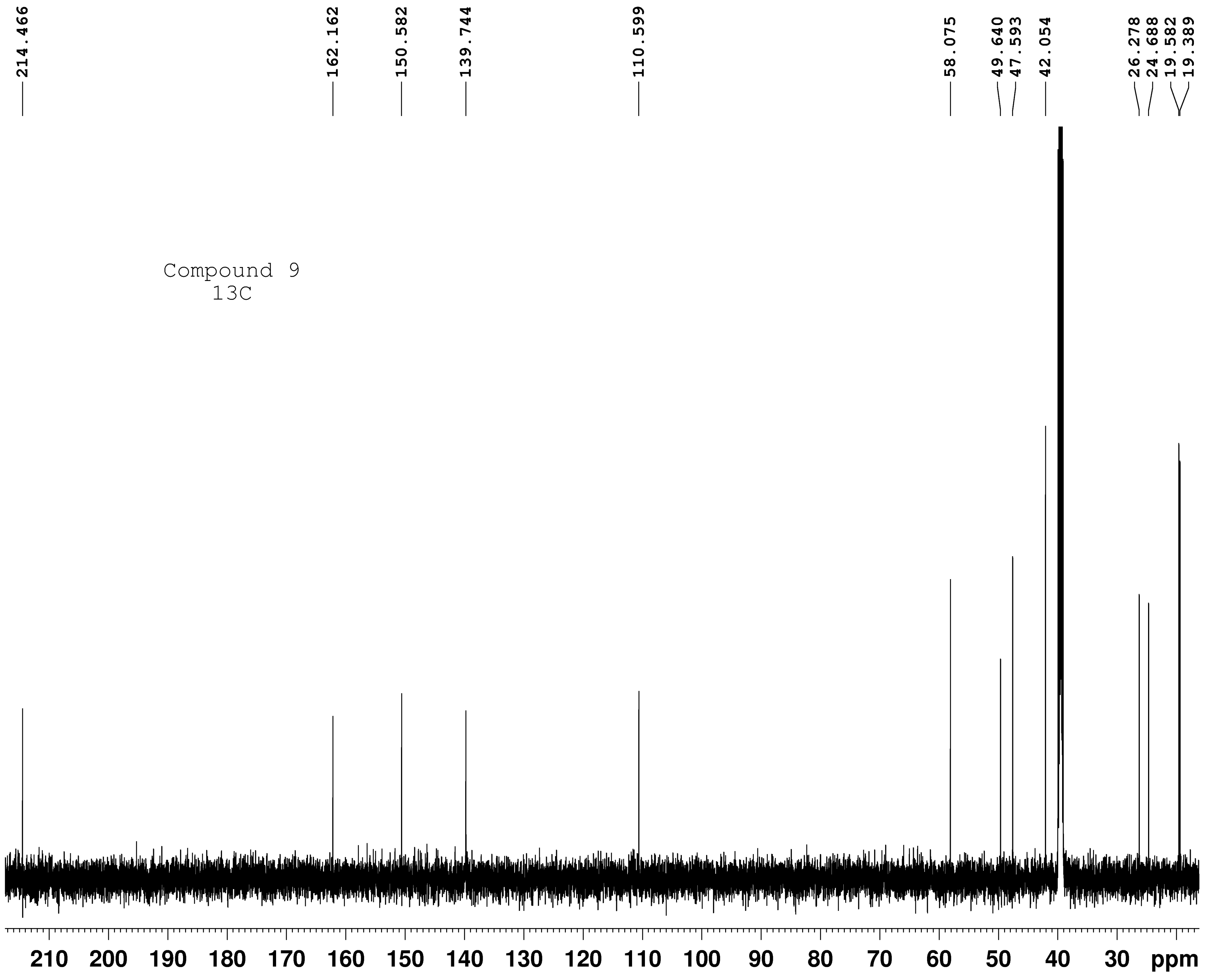

NAME	DN-98A	
EXPNO	12	
PROCNO	1	
Date_	20180629	
Time	15.02	h
INSTRUM	spect	
PROBHD	Z847801_0047 (	
PULPROG	zgdc30	
TD	32768	
SOLVENT	DMSO	
NS	128	
DS	0	
SWH	36057.691	Hz
FIDRES	2.200787	Hz
AQ	0.4544329	sec
RG	2050	
DW	13.867	usec
DE	6.50	usec
TE	293.0	K
D1	1.50000000	sec
D11	0.03000000	sec
TD0	1	
SFO1	150.8892338	MHz
NUC1	13C	
P1	9.80	usec
SI	65536	
SF	150.8726997	MHz
WDW	EM	
SSB	0	
LB	1.00	Hz
GB	0	
PC	1.40	

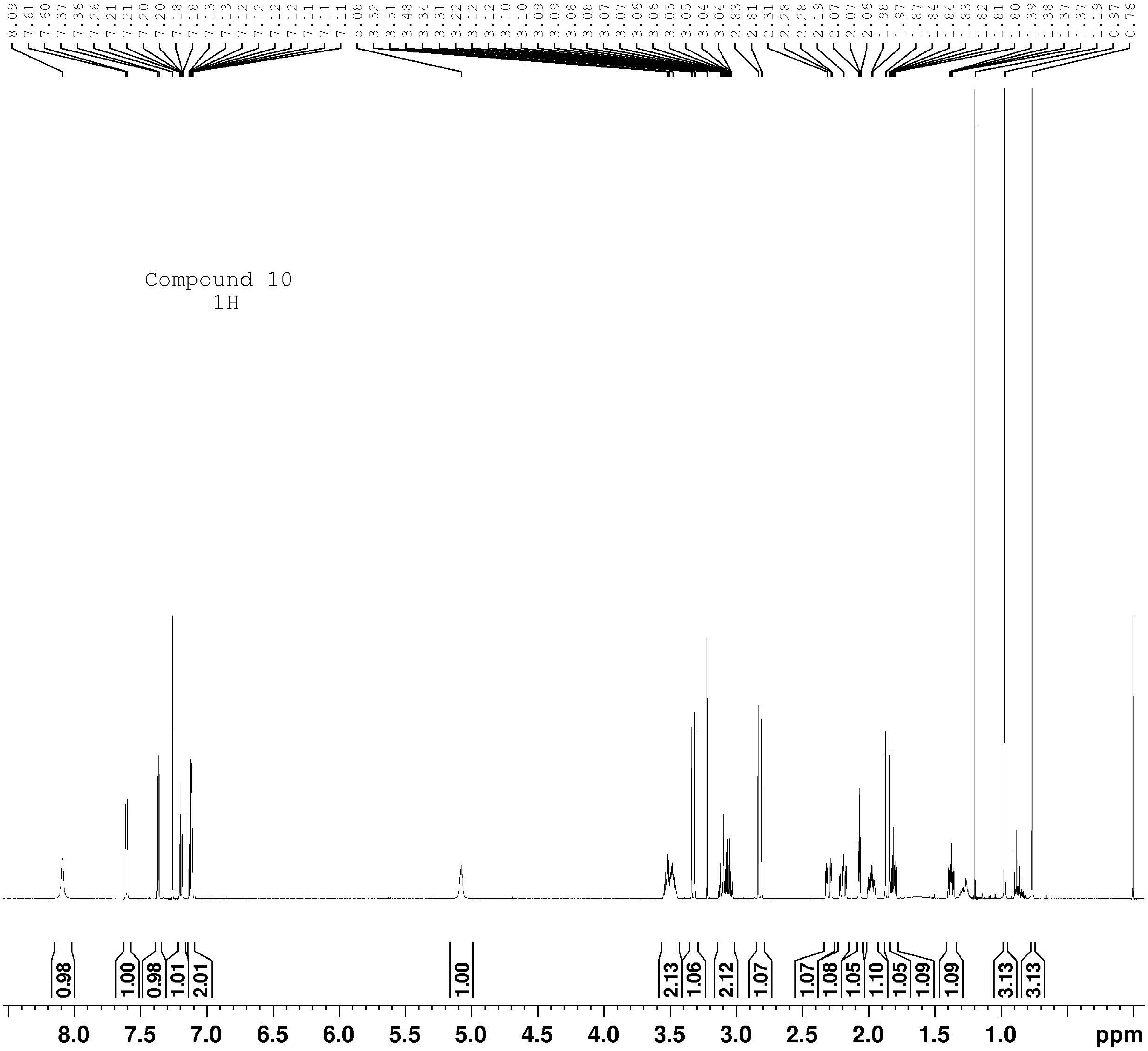

Current Data Parameters

NAME	DN-102A
EXPNO	21
PROCNO	1

F2 - Acquisition Parameters

Date_	20180706
Time	15.46 h
INSTRUM	spect
PROBHD	Z847801_0047 (
PULPROG	zg30
TD	32768
SOLVENT	CDCl3
NS	16
DS	0
SWH	9615.385 Hz
FIDRES	0.586877 Hz
AQ	1.7039360 sec
RG	203
DW	52.000 usec
DE	13.95 usec
TE	293.0 K
D1	1.00000000 sec
TD0	1
SFO1	600.0145608 MHz
NUC1	1H
P1	10.85 usec
PLW1	20.00000000 W

F2 - Processing parameters

SI	65536
SF	600.0100156 MHz
WDW	EM
SSB	0
LB	0 Hz
GB	0
PC	1.00

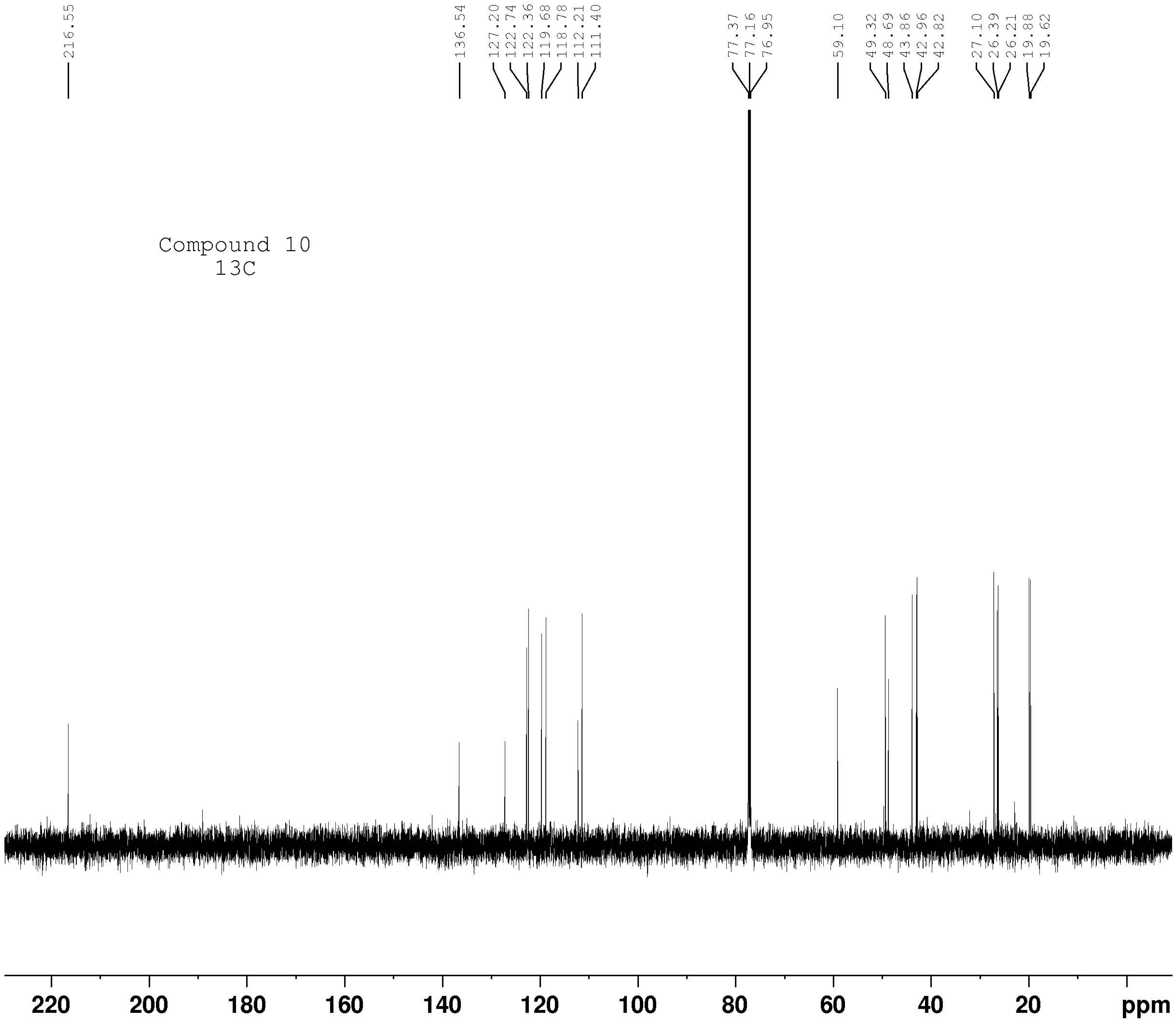

Current Data Parameters
NAME DN-102A
EXPNO 22
PROCNO 1

F2 - Acquisition Parameters
Date_ 20180706
Time 15.51 h
INSTRUM spect
PROBHD Z847801_0047 (
PULPROG zgdc30
TD 32768
SOLVENT CDCl3
NS 128
DS 0
SWH 36057.691 Hz
FIDRES 2.200787 Hz
AQ 0.4543829 sec
RG 2050
DW 13.867 usec
DE 6.50 usec
TE 293.1 K
D1 1.50000000 sec
D11 0.03000000 sec
TD0 1
SFO1 150.8892338 MHz
NUC1 13C
P1 9.80 usec
PLW1 40.00000000 W
SFO2 600.0124004 MHz
NUC2 1H
CPDPRG[2 waltz16
PCPD2 90.00 usec
PLW2 20.00000000 W
PLW12 0.33800000 W

F2 - Processing parameters
SI 65536
SF 150.8726181 MHz
WDW EM
SSB 0
LB 1.00 Hz
GB 0
PC 1.40

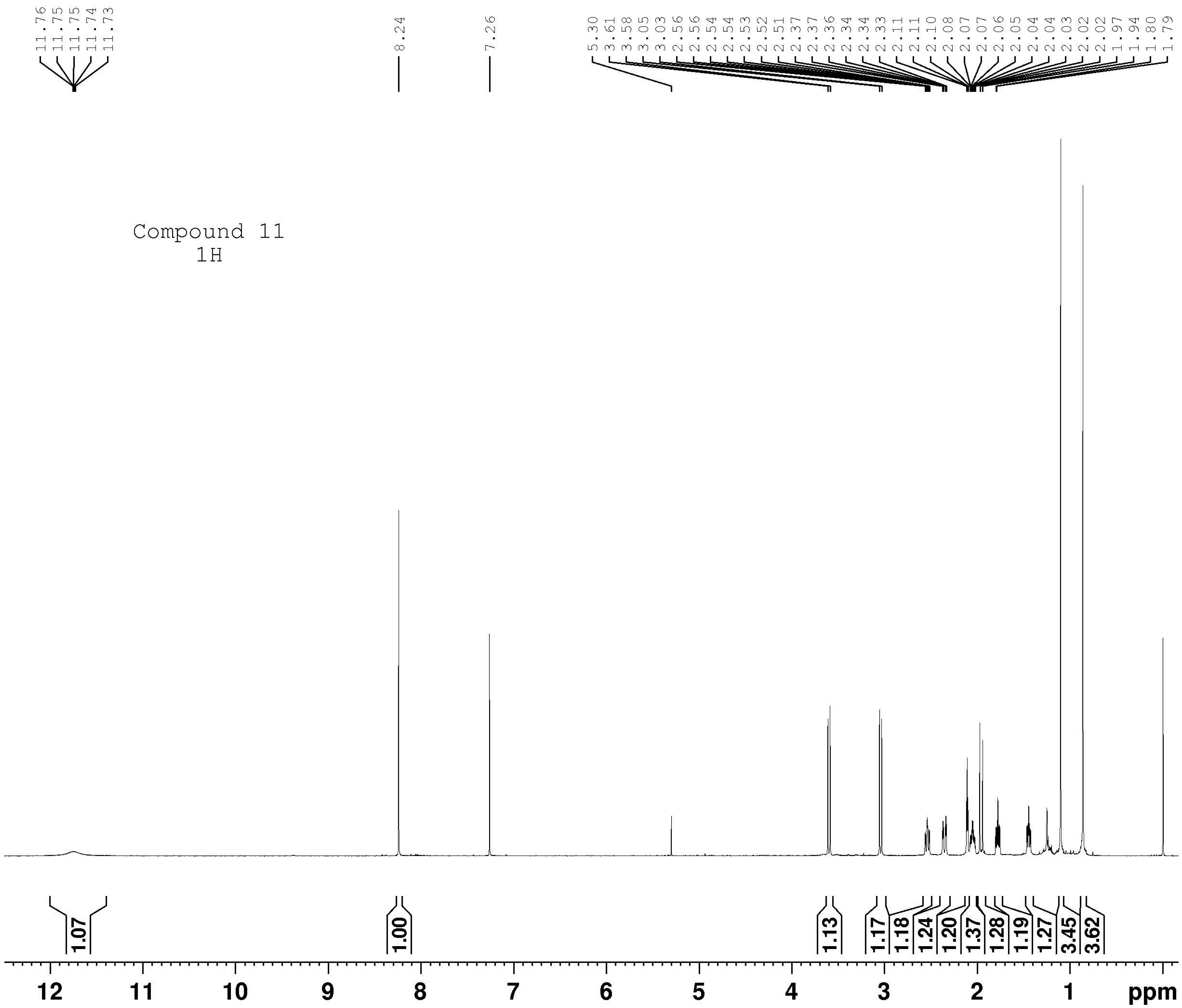

Current Data Parameters

NAME	DN-108A	
EXPNO	11	
PROCNO	1	

F2 - Acquisition Parameters

Date_	20180717	
Time	14.30	h
INSTRUM	spect	
PROBHD	Z847801_0047 (	
PULPROG	zg30	
TD	32768	
SOLVENT	CDCl3	
NS	16	
DS	0	
SWH	9615.385	Hz
FIDRES	0.586877	Hz
AQ	1.7039360	sec
RG	362	
DW	52.000	usec
DE	13.95	usec
TE	293.0	K
D1	1.00000000	sec
TD0	1	
SFO1	600.0145608	MHz
NUC1	1H	
P1	10.85	usec
PLW1	20.00000000	W

F2 - Processing parameters

SI	65536	
SF	600.0100152	MHz
WDW	no	
SSB	0	
LB	0	Hz
GB	0	
PC	1.00	

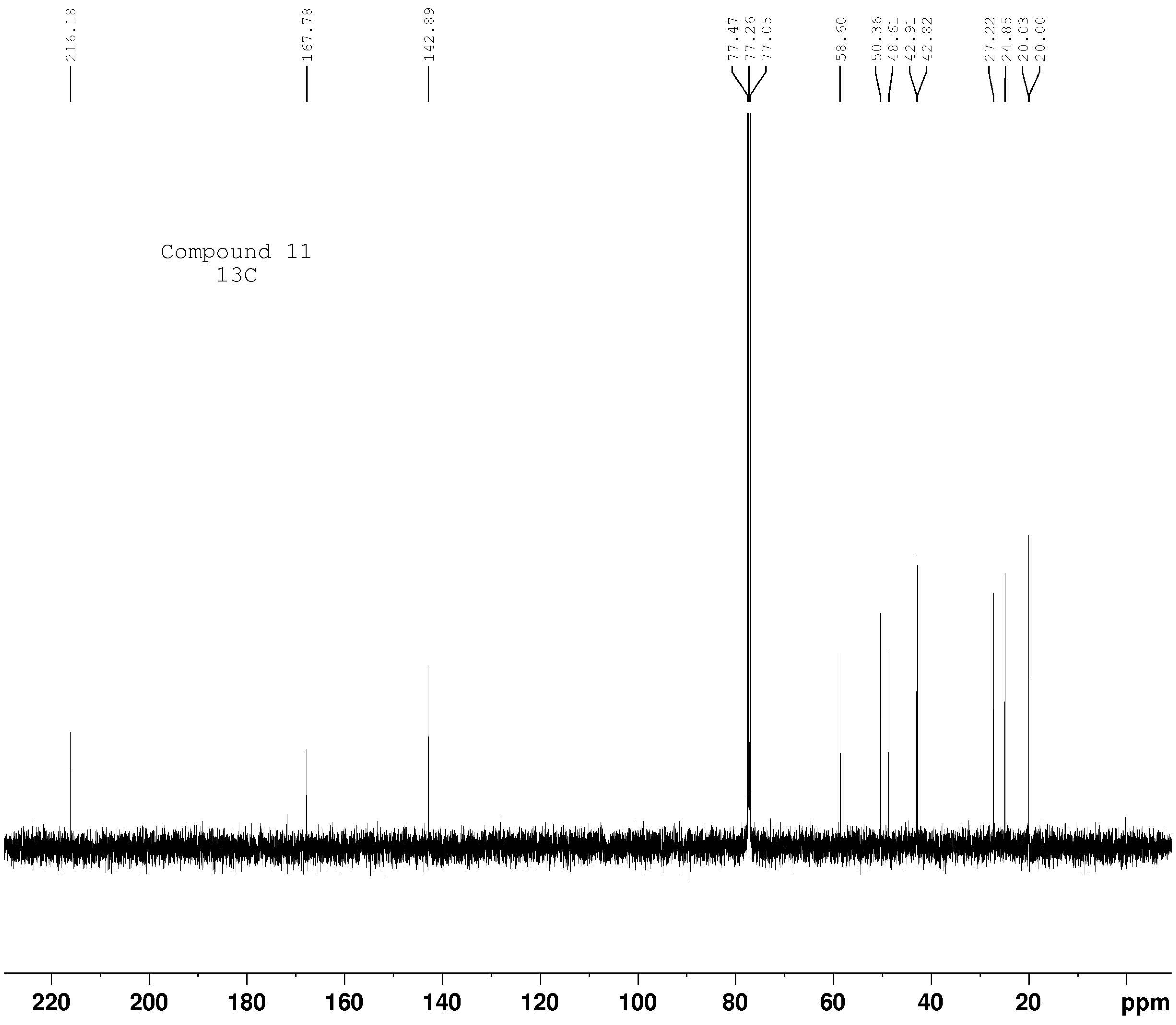

Current Data Parameters

NAME	DN-108A
EXPNO	12
PROCNO	1

F2 - Acquisition Parameters

Date_	20180717
Time	14.47 h
INSTRUM	spect
PROBHD	Z847801_0047 (
PULPROG	zgdc30
TD	32768
SOLVENT	CDCl3
NS	256
DS	0
SWH	36057.691 Hz
FIDRES	2.200787 Hz
AQ	0.4543829 sec
RG	2050
DW	13.867 usec
DE	6.50 usec
TE	293.3 K
D1	1.50000000 sec
D11	0.03000000 sec
TD0	1
SFO1	150.8892338 MHz
NUC1	13C
P1	9.80 usec
PLW1	40.00000000 W
SFO2	600.0124004 MHz
NUC2	1H
CPDPRG[2	waltz16
PCPD2	90.00 usec
PLW2	20.00000000 W
PLW12	0.33800000 W

F2 - Processing parameters

SI	65536
SF	150.8726021 MHz
WDW	EM
SSB	0
LB	1.00 Hz
GB	0
PC	1.40

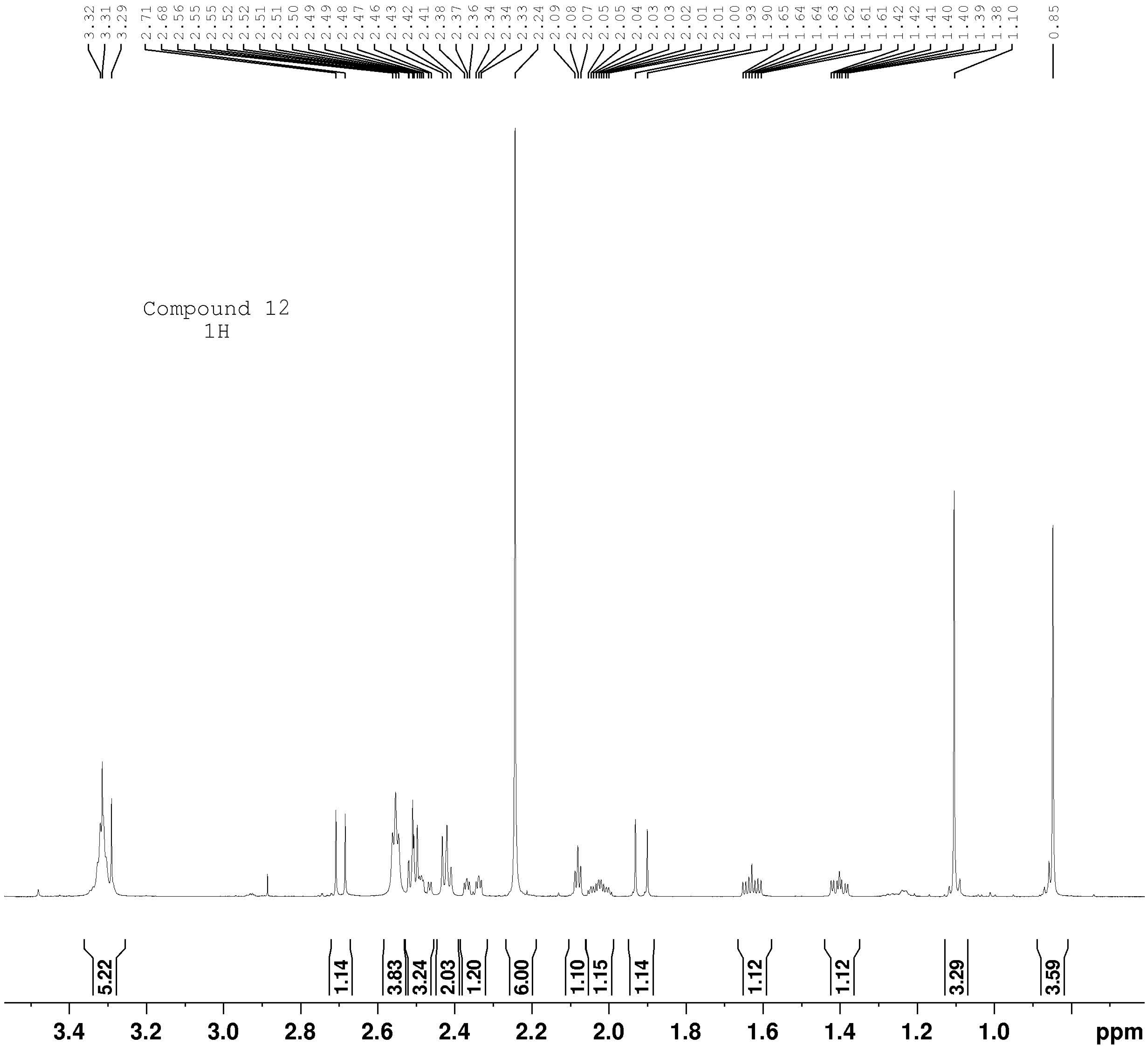

Current Data Parameters
NAME CC-76A
EXPNO 11
PROCNO 1

F2 - Acquisition Parameters

Date_	20190218	
Time	19.23	h
INSTRUM	spect	
PROBHD	Z847801_0047 (	
PULPROG	zg30	
TD	32768	
SOLVENT	CDCl3	
NS	1	
DS	0	
SWH	9615.385	Hz
FIDRES	0.586877	Hz
AQ	1.7039360	sec
RG	50.8	
DW	52.000	usec
DE	13.95	usec
TE	293.0	K
D1	1.00000000	sec
TD0	1	
SFO1	600.0145608	MHz
NUC1	1H	
P1	10.85	usec
PLW1	20.00000000	W

F2 - Processing parameters

SI	65536	
SF	600.0100148	MHz
WDW	EM	
SSB	0	
LB	0	Hz
GB	0	
PC	1.00	

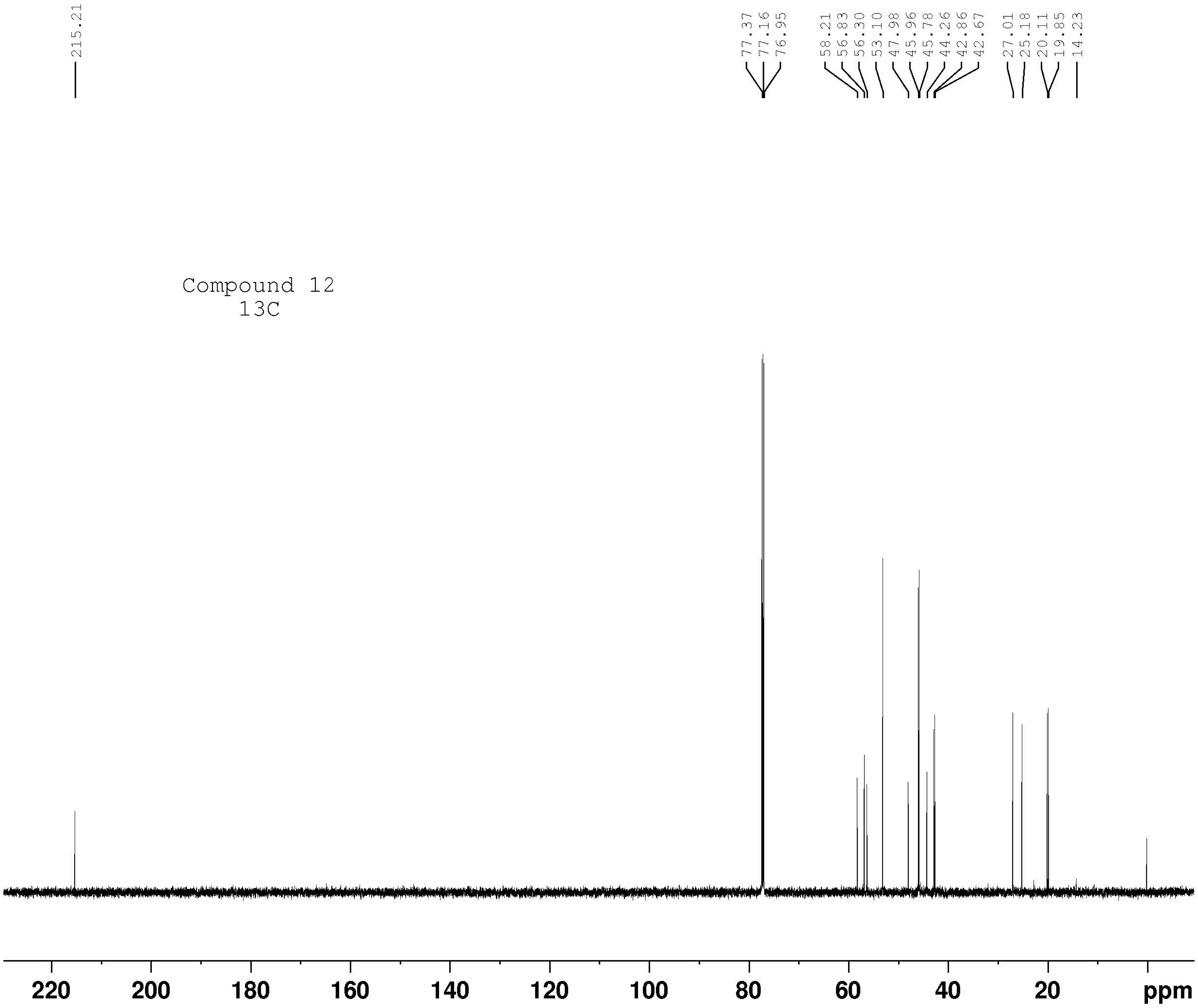

Current Data Parameters
NAME CC-76A
EXPNO 16
PROCNO 1

F2 - Acquisition Parameters
Date_ 20190218
Time 19.57 h
INSTRUM spect
PROBHD Z847801_0047 (
PULPROG zgdc30
TD 32768
SOLVENT CDCl3
NS 128
DS 0
SWH 36057.691 Hz
FIDRES 2.200787 Hz
AQ 0.4543829 sec
RG 2050
DW 13.867 usec
DE 6.50 usec
TE 293.0 K
D1 1.50000000 sec
D11 0.03000000 sec
TD0 1
SFO1 150.8892338 MHz
NUC1 13C
P1 9.80 usec
PLW1 40.00000000 W
SFO2 600.0124004 MHz
NUC2 1H
CPDPRG[2 waltz16
PCPD2 90.00 usec
PLW2 20.00000000 W
PLW12 0.33800000 W

F2 - Processing parameters
SI 65536
SF 150.8726216 MHz
WDW EM
SSB 0
LB 1.00 Hz
GB 0
PC 1.40

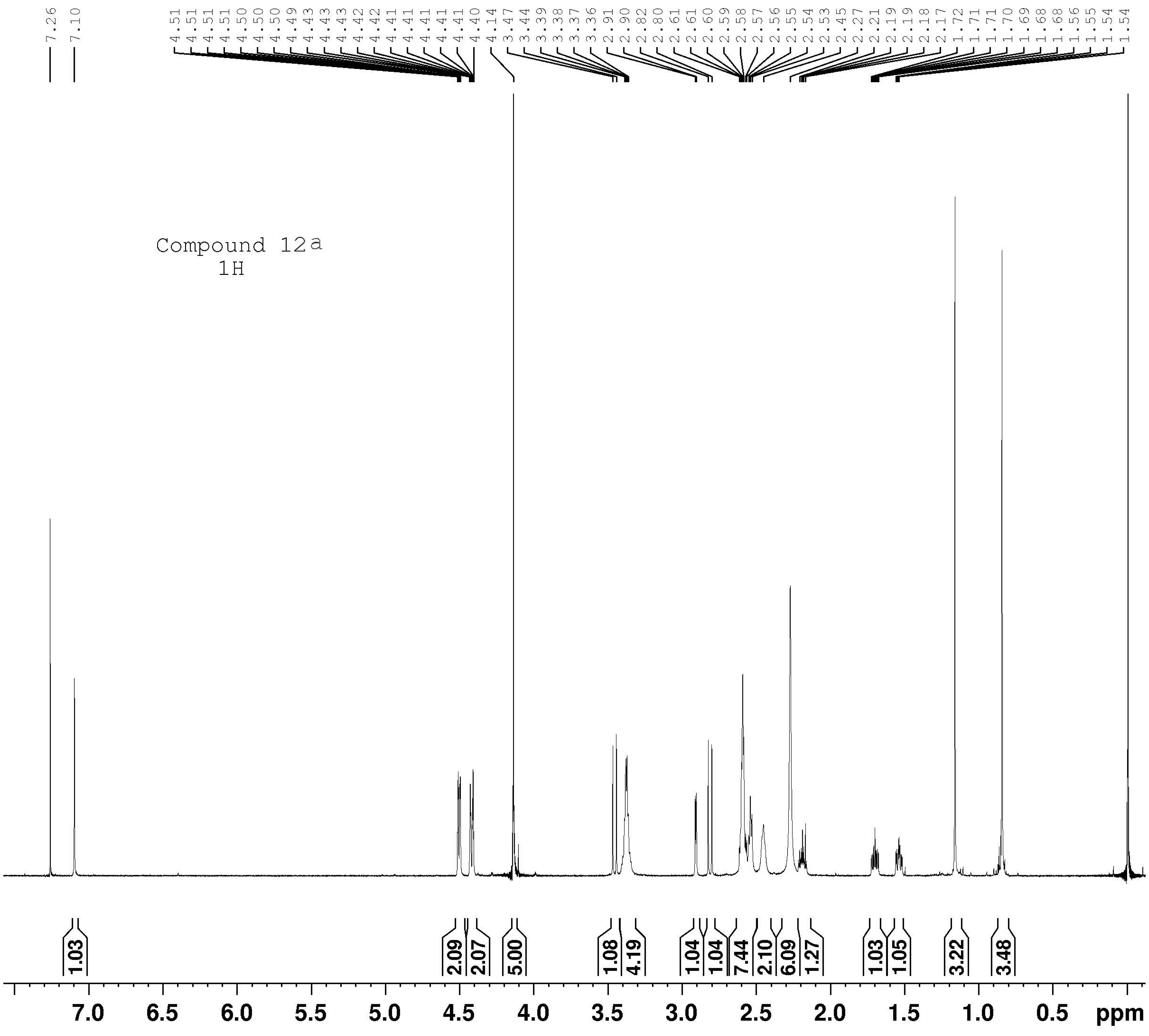

Current Data Parameters
NAME CC-78A
EXPNO 11
PROCNO 1

F2 - Acquisition Parameters
Date_ 20190218
Time 17.55 h
INSTRUM spect
PROBHD Z847801_0047 (
PULPROG zg30
TD 32768
SOLVENT CDCl3
NS 1
DS 0
SWH 9615.385 Hz
FIDRES 0.586877 Hz
AQ 1.7039360 sec
RG 144
DW 52.000 usec
DE 13.95 usec
TE 293.0 K
D1 1.00000000 sec
TD0 1
SFO1 600.0145608 MHz
NUC1 1H
P1 10.85 usec
PLW1 20.00000000 W

F2 - Processing parameters
SI 65536
SF 600.0100152 MHz
WDW EM
SSB 0
LB 0 Hz
GB 0
PC 1.00

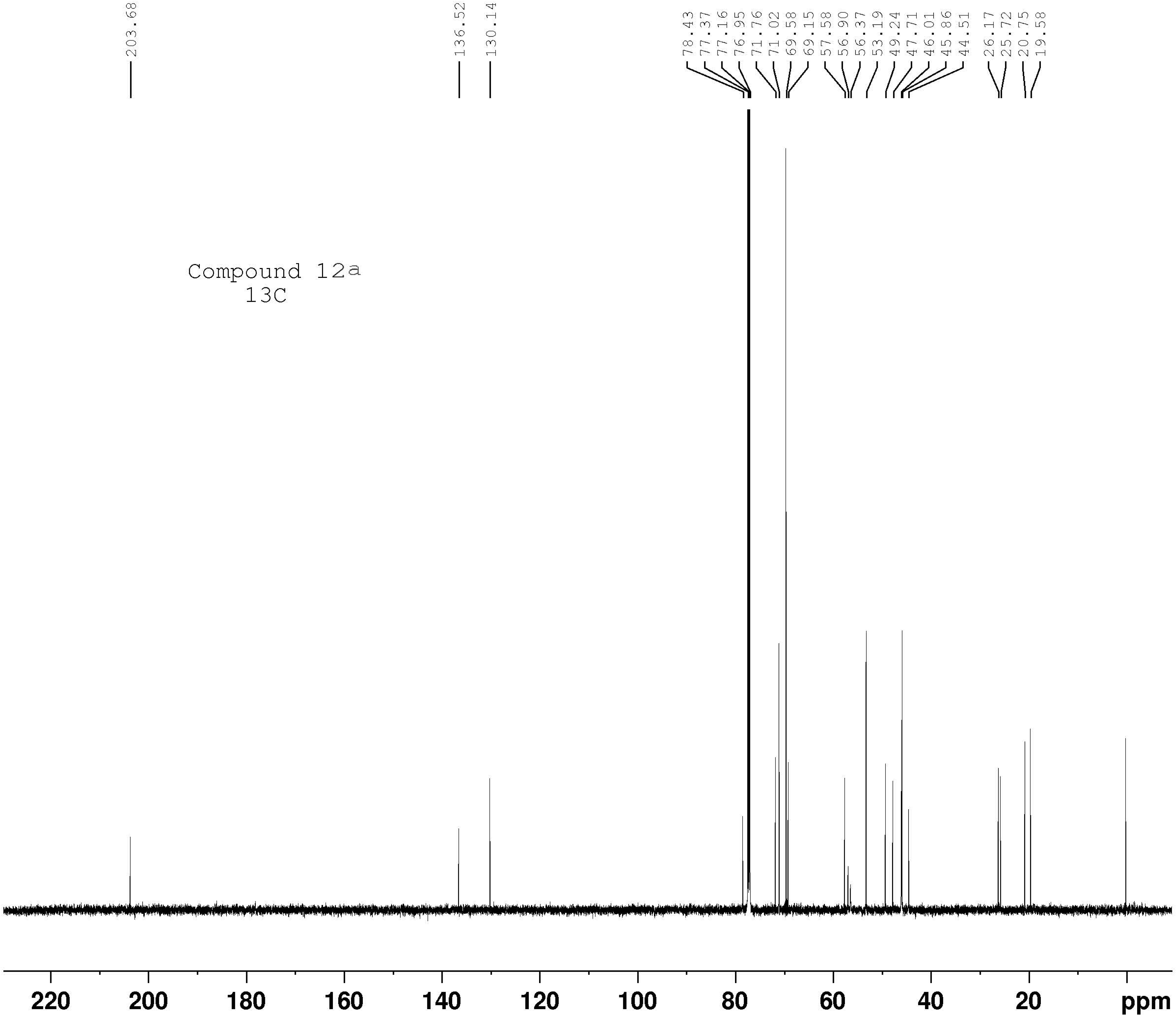

Current Data Parameters

NAME	CC-78A
EXPNO	16
PROCNO	1

F2 - Acquisition Parameters

Date_	20190218
Time	19.11 h
INSTRUM	spect
PROBHD	Z847801_0047 (
PULPROG	zgdc30
TD	32768
SOLVENT	CDCl3
NS	1024
DS	0
SWH	36057.691 Hz
FIDRES	2.200787 Hz
AQ	0.4543829 sec
RG	2050
DW	13.867 usec
DE	6.50 usec
TE	293.0 K
D1	1.50000000 sec
D11	0.03000000 sec
TD0	1
SFO1	150.8892338 MHz
NUC1	13C
P1	9.80 usec
PLW1	40.00000000 W
SFO2	600.0124004 MHz
NUC2	1H
CPDPRG[2	waltz16
PCPD2	90.00 usec
PLW2	20.00000000 W
PLW12	0.33800000 W

F2 - Processing parameters

SI	65536
SF	150.8726180 MHz
WDW	EM
SSB	0
LB	1.00 Hz
GB	0
PC	1.40

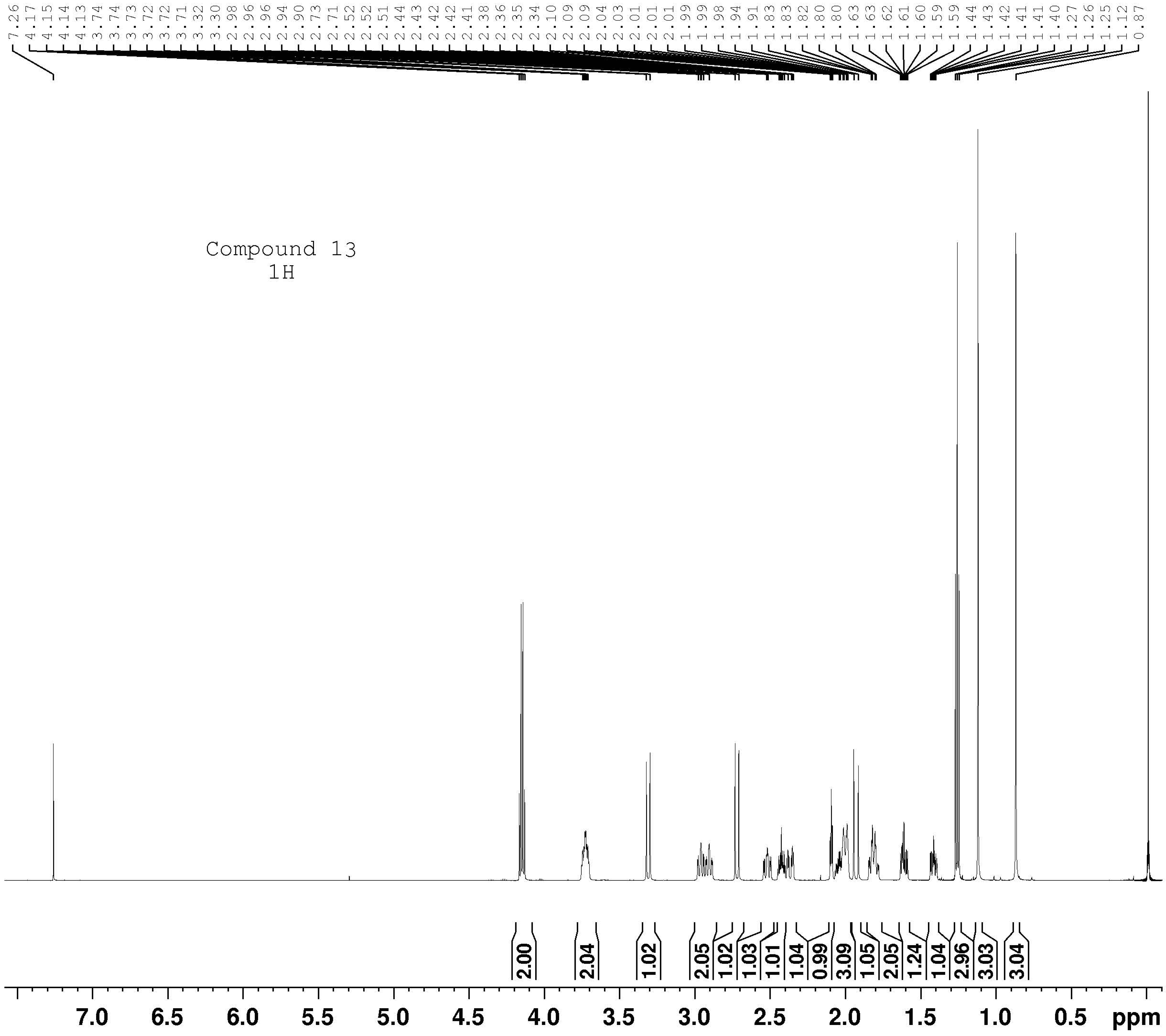

Current Data Parameters

NAME	CD-03A
EXPNO	11
PROCNO	1

F2 - Acquisition Parameters

Date_	20190225
Time	21.02 h
INSTRUM	spect
PROBHD	Z847801_0047 (
PULPROG	zg30
TD	32768
SOLVENT	CDCl3
NS	32
DS	0
SWH	9615.385 Hz
FIDRES	0.586877 Hz
AQ	1.7039360 sec
RG	101
DW	52.000 usec
DE	13.95 usec
TE	293.0 K
D1	1.00000000 sec
TD0	1
SFO1	600.0145608 MHz
NUC1	1H
P1	10.85 usec
PLW1	20.00000000 W

F2 - Processing parameters

SI	65536
SF	600.0100155 MHz
WDW	EM
SSB	0
LB	0 Hz
GB	0
PC	1.00

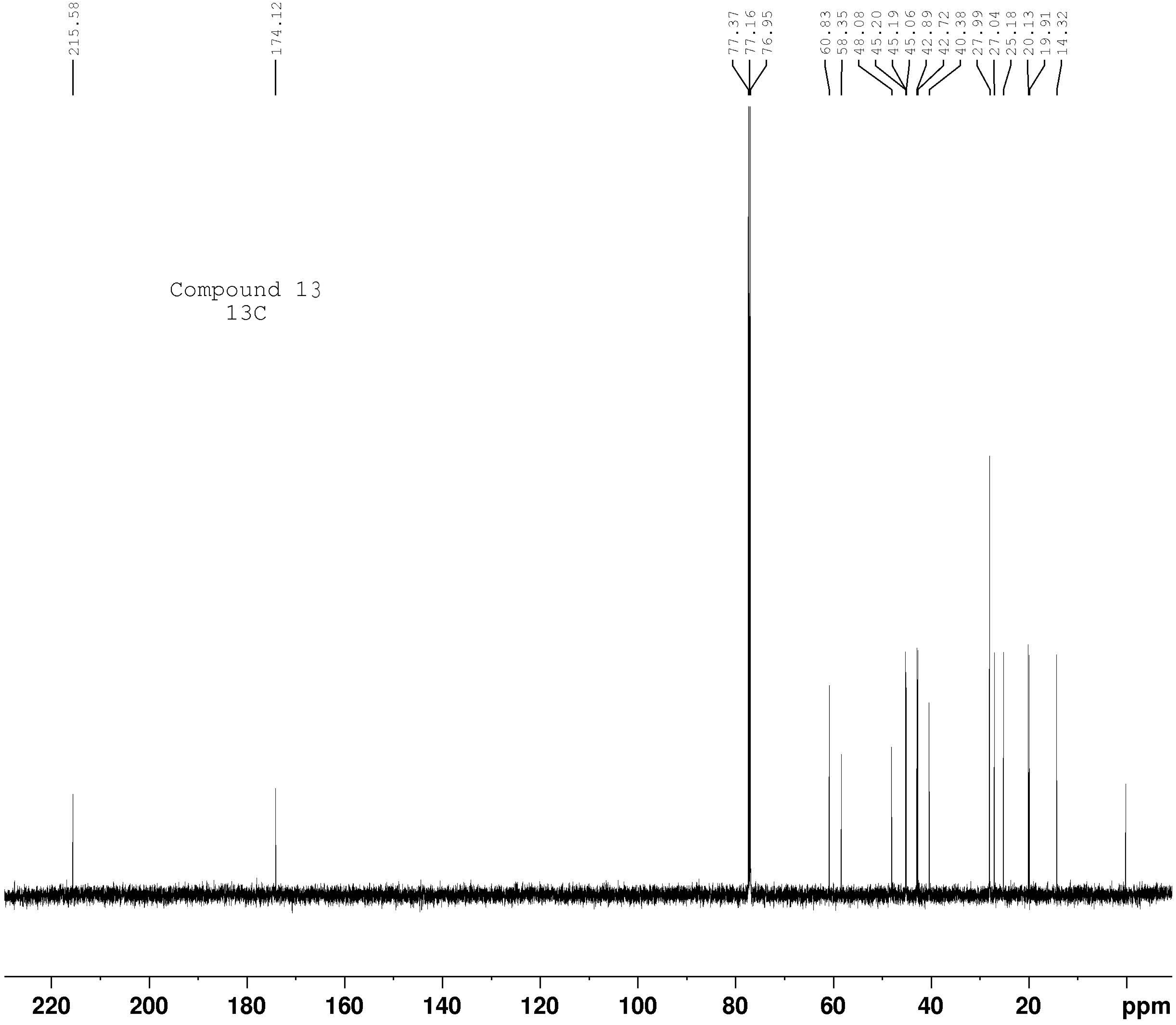


```
Current Data Parameters
NAME              CD-03A
EXPNO                 16
PROCNO                 1

F2 - Acquisition Parameters
Date_           20190225
Time               21.48 h
INSTRUM            spect
PROBHD   Z847801_0047 (
PULPROG           zgdc30
TD                 32768
SOLVENT            CDCl3
NS                   128
DS                     0
SWH            36057.691 Hz
FIDRES          2.200787 Hz
AQ             0.4543829 sec
RG                  2050
DW                13.867 usec
DE                  6.50 usec
TE                 293.0 K
D1            1.50000000 sec
D11           0.03000000 sec
TD0                    1
SFO1         150.8892338 MHz
NUC1                 13C
P1                  9.80 usec
PLW1         40.00000000 W
SFO2         600.0124004 MHz
NUC2                  1H
CPDPRG[2         waltz16
PCPD2              90.00 usec
PLW2         20.00000000 W
PLW12         0.33800000 W

F2 - Processing parameters
SI                 65536
SF           150.8726187 MHz
WDW                   EM
SSB                    0
LB                  1.00 Hz
GB                     0
PC                  1.40
```
